# Increased respiratory morbidity in individuals with interstitial lung abnormalities

**DOI:** 10.1186/s12890-020-1107-0

**Published:** 2020-03-19

**Authors:** Nils Hoyer, Laura H. Thomsen, Mathilde M. W. Wille, Torgny Wilcke, Asger Dirksen, Jesper H. Pedersen, Zaigham Saghir, Haseem Ashraf, Saher B. Shaker

**Affiliations:** 1 Department of Respiratory Medicine, Herlev and Gentofte Hospital, Kildegårdsvej 28, 2900 Hellerup, Copenhagen, Denmark; 2 Department of Respiratory Medicine, Amager and Hvidovre Hospital, Copenhagen, Denmark; 3 Department of Radiology, North Zealand Hospital, Hillerød, Denmark; 4 Department of Cardiothoracic Surgery RT, Copenhagen University Hospital Rigshospitalet, Copenhagen, Denmark; 5 Department of Radiology, Akershus University Hospital, Loerenskog, Norway; 6 Division of Medicine and Laboratory Sciences, University of Oslo, Oslo, Norway

**Keywords:** Interstitial fibrosis, Lung Cancer, Clinical epidemiology, Imaging

## Abstract

**Background:**

Interstitial lung abnormalities (ILA) are common in participants of lung cancer screening trials and broad population-based cohorts. They are associated with increased mortality, but less is known about disease specific morbidity and healthcare utilisation in individuals with ILA.

**Methods:**

We included all participants from the screening arm of the Danish Lung Cancer Screening Trial with available baseline CT scan data (*n* = 1990) in this cohort study. The baseline scan was scored for the presence of ILA and patients were followed for up to 12 years. Data about all hospital admissions, primary healthcare visits and medicine prescriptions were collected from the Danish National Health Registries and used to determine the participants’ disease specific morbidity and healthcare utilisation using Cox proportional hazards models.

**Results:**

The 332 (16.7%) participants with ILA were more likely to be diagnosed with one of several respiratory diseases, including interstitial lung disease (HR: 4.9, 95% CI: 1.8–13.3, *p* = 0.008), COPD (HR: 1.7, 95% CI: 1.2–2.3, *p* = 0.01), pneumonia (HR: 2.0, 95% CI: 1.4–2.7, *p* <  0.001), lung cancer (HR: 2.7, 95% CI: 1.8–4.0, *p* <  0.001) and respiratory failure (HR: 1.8, 95% CI: 1.1–3.0, *p* = 0.03) compared with participants without ILA. These findings were confirmed by increased hospital admission rates with these diagnoses and more frequent prescriptions for inhalation medicine and antibiotics in participants with ILA.

**Conclusions:**

Individuals with ILA are more likely to receive a diagnosis and treatment for several respiratory diseases, including interstitial lung disease, COPD, pneumonia, lung cancer and respiratory failure during long-term follow-up.

## Introduction

Interstitial lung abnormalities (ILA) are a group of radiological findings visible on computed tomography (CT) of the lung in individuals without a diagnosis of interstitial lung disease (ILD) [1]. They are common in participants of lung cancer screening trials, patients with chronic obstructive pulmonary disease (COPD) and broad population-based cohorts [2–8]. Similar radiologic findings (nodular changes, reticulation, ground glass opacities and honeycombing) are also present in several interstitial lung diseases (ILDs), including idiopathic pulmonary fibrosis (IPF), and can precede onset of disease symptoms and diagnosis by several years [1, 9, 10].

ILA are associated with increased mortality as well as reduced lung volumes and exercise capacity [6, 7, 11]. In addition, radiologic progression of ILA has been shown in longitudinal studies and it has been suggested that ILA represent subclinical ILD in some patients [1, 2, 4, 8, 12]. There are reports of a higher mortality from lung cancer and pulmonary fibrosis in patients with ILA [7, 13]. However, the prevalence of ILA is much higher than the prevalence of ILDs as clinical entities and only a few of all people with ILA develop clinical disease [7, 13–16]. The role of ILA in the early detection of ILD remains to be established.

Several ILDs are known to lead to an increase in healthcare utilisation, including diagnostic procedures, hospital admissions, emergency department visits, medical treatment and lung transplantations [17–19]. However, data about the development of specific diseases and healthcare utilisation in individuals with ILA are still limited.

The objective of this study was to investigate the association between incidental findings of ILA and disease specific morbidity such as the diagnosis of ILD and other diseases, hospital admission rates, primary care contacts and medicine use.

## Methods

### Study population

This registry-based follow-up cohort study included all participants from the intervention arm of the Danish Lung Cancer Screening Trial (DLCST) with available CT scan data (*n* = 1990) (Supplementary Figure 1). Methods of the DLCST, including criteria of eligibility, have been published previously and are briefly detailed below [20]. The DLCST was a 4-year, 5-round prospective randomized controlled screening trial. From the year 2004, 4104 participants aged 50–75 years with a smoking history of at least 20 pack-years were recruited by newspaper advertisements [20]. All participants were active or former smokers. If participants were former smokers, they had to have quit after the age of 50 years and within the previous 10 years. The inclusion criteria included an FEV_1_ value of at least 30% of the predicted value at baseline and participants had to be able to climb two flights of stairs (total of 36 steps) without pausing [20]. Exclusion criteria were the following: weight over 130 kg, history of cancer diagnosis and treatment, lung tuberculosis, shortened life expectancy less than 10 years (according to the judgement of the recruiting physician), and chest CT performed during the past year for any reason [20]. Spirometry was performed by professionally trained and experienced pulmonary function technicians or nurses, and were expressed in absolute values and as a percentage of predicted values according to European reference equations [20, 21].

### Imaging and image review

Details about the imaging procedure have previously been published and are briefly described below [20, 22]. Examination of the screening group used a multi-slice CT system (16 rows Philips Mx 8000, Philips Medical Systems) performed supine at full inspiration with a low-dose technique (120 kV and 40mAs). Thin slices were reconstructed with a hard algorithm before visual assessment. Two different sets of all scans in random order were created before evaluation by two independent observers (LHT and MMWW) who were blinded to person identification and date of scan. Interstitial lung abnormalities were registered as either absent or present. If present, ILA were categorized as centrilobular, pleural, or paraseptal nodules, ground-glass attenuation, reticulation or honeycombing (Supplementary Table 3) [22]. The interobserver agreement in the detection of ILA was fair to nearly substantial (kappa 0.60) and has previously been published in more detail [22]. Statistical analyses were performed on a combined ILA variable, classifying participants as having ILA if at least one observer noted a finding of ILA.

### Registries

Registry data for all participants were obtained from the Danish national health registries covering the entire population. Data on public and private hospital admissions, outpatient clinic visits, emergency department visits including the diagnoses for these contacts were obtained from the Danish National Patient Register. Data on visits to a primary care provider were obtained from the Danish National Health Insurance Service Register. Data on medicine use were obtained from the Danish Prescription Database. Patients were followed up until May 5, 2016.

### Data analysis

Analysis of baseline characteristics was performed with an unpaired t-test or Fisher’s exact test for continuous and categorical variables, respectively.

Analysis of the association between ILA and the development of disease was based on all registered diagnoses (the primary discharge diagnosis and contributing or underlying diagnoses) for hospital admissions, outpatient clinic visits or emergency department visits. The ICD10-codes used to define the specific diseases and disease groups are listed in Supplementary Table 1. We used Cox regression analysis to handle the censoring that was introduced by the known increased mortality of participants with ILA [13].

Analysis of the association between ILA and hospital admission rates was exclusively based on the primary discharge diagnosis of hospital admissions to avoid overestimating chronic diseases that would often be listed as contributing diagnoses. Because of the possibility of repeated admissions with the same diagnosis, we used recurrent event Cox regression analysis using an Andersen-Gill model with death included as a censoring event. To adjust for within-subject correlation, the model included a count of previous admissions as a covariate.

The multivariate Cox regression models were adjusted for age, sex, BMI and pack-years. An extended model which also included baseline measurements of FEV_1_ was also used for the analysis of disease development (Supplementary Table 4). For the analyses of development of disease and hospital admission rates the proportional hazards assumptions of the Cox models were violated by pack-years and body mass index (BMI), which consequently were included as stratifying variables in the models, allowing for varying hazard functions. Cumulative event curves of expected hospital admissions are based on a marginal model of a recurrent event Cox proportional hazards model with gap times (patients were not at risk for admission while hospitalised), and a terminal event (death) model [23]. To control the false discovery rate that could result from multiple comparisons, we applied the Benjamini-Hochberg procedure to all *p*-values.

The associations between ILA and primary care visits or prescription medicine use were determined by negative binomial regression analysis, using observation time as offset to adjust for a shortened observation time caused by the increased mortality of participants with ILA [13]. The models were adjusted for age, sex, BMI and pack-years. The ATC codes used to classify prescription medicine are listed in Supplementary Table 2.

Two-sided *p*-values below 0.05 were considered significant. Missing data were handled by listwise deletion without imputation of data. All statistical analyses were performed with the statistical software R (version 3.5.1).

## Results

The 332 (16.7%) participants of the Danish Lung Cancer Screening Trial with ILA were older, had lower FEV_1_% predicted and FVC % predicted, and had more frequently airway obstruction (FEV_1_/FVC <  0.7) at the time of their CT scan, compared with participants without ILA (Table 1).

**Table 1:** Baseline characteristics of participants with or without a baseline CT finding of ILA

	ILA (*n* = 332)	No ILA (*n* = 1658)	*p*-value
Age mean (SD), years	59.7 (5.0)	57.6 (4.7)	< 0.001
Female, n (%)	136 (41%)	742 (45%)	0.20
BMI, mean (SD)	24.9 (4.0)	25.3 (3.8)	0.06
Current/former smokers, n (%)	256/76 (77%/23%)	1243/415 (74%/26%)	0.41
Pack-years, mean (SD)	37.5 (13.4)	36.18 (13.4)	0.10
FEV_1_ l, mean (SD)	2.75 (0.76)	2.91 (0.75)	< 0.001
FEV_1_% predicted, mean (SD)	87.9 (18.6)	92.4 (16.3)	< 0.001
FVC l, mean (SD)	4.03(1.0)	4.13 (0.99)	0.10
FVC % predicted, mean (SD)	99.5 (17.7)	101.7 (15.3)	0.04
FEV_1_/FVC < 0.7, n (%)	178 (54%)	693 (42%)	0.001
Follow-up time median, years (IQR)	11.22 (10.77–11.75)	11.29 (11.03–11.75)	< 0.001

*ILA* interstitial lung abnormalities, *SD* standard deviation, *BMI* body mass index, *FEV*_*1*_ forced expiratory volume in one second, *FVC* forced vital capacity, *IQR* interquartile range

### ILA and specific diagnoses

**Fig. 1:**
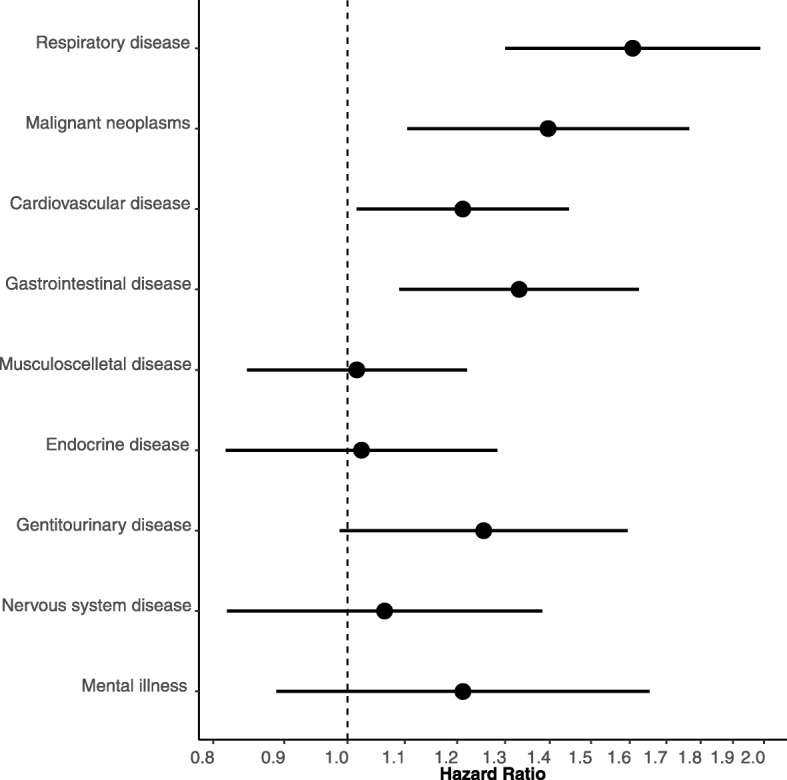
Hazard ratio for participants with ILA compared with those without ILA for receiving a hospital diagnosis within a specific diagnostic group. The multivariate Cox regression analysis is adjusted for age, sex, BMI and pack-years. Error bars represent 95% confidence intervals

In multivariate Cox proportional hazards analysis, participants with ILA were more likely to be diagnosed with one of several respiratory, malignant or cardiovascular diseases compared with those without ILA (Table 2, Fig. 1). Respiratory diseases were most markedly increased, including COPD, pneumonia, pleural empyema or lung abscess, ILD and respiratory failure (Table 2). Moreover, we found an increase in gastrointestinal disease, which was driven by an increase in functional intestinal disorders (Table 2, Fig. 1).

**Table 2:** Specific diagnoses prompting hospital admissions, outpatient clinic visits or emergency department visits

Diagnosis	ILA (%)	No ILA (%)	HR	95% CI	Adjusted *p*-value
Respiratory	116 (34.9)	361 (21.8)	1.6	1.3–2.0	< 0.001
COPD	55 (16.6)	159 (9.6)	1.7	1.2–2.3	0.01
Pneumonia	56 (16.9)	126 (7.6)	2.0	1.4–2.7	< 0.001
Asthma	4 (1.2)	47 (2.8)	0.4	0.2–1.2	0.15
Pleural empyema or lung abscess	5 (1.5)	3 (0.2)	6.6	1.5–28.8	0.03
Interstitial lung disease	8 (2.4)	8 (0.5)	4.9	1.8–13.3	0.008
Respiratory failure	25 (7.5)	61 (3.7)	1.8	1.1–3.0	0.03
Malignant neoplasm	93 (28.0)	317 (19.1)	1.4	1.1–1.8	0.02
Lung cancer	39 (11.7)	71 (4.3)	2.7	1.8–4.0	< 0.001
Non-pulmonary cancer	67 (20.2)	266 (16.0)	1.2	0.9–1.6	0.30
Cardiovascular	157 (47.3)	652 (39.3)	1.2	1.0–1.5	0.09
Heart failure	19 (5.7)	47 (2.8)	1.7	1.0–3.0	0.19
Pulmonary embolism	8 (2.4)	22 (1.3)	1.9	0.8–4.3	0.17
Atrial fibrillation/atrial flutter	27 (8.1)	95 (5.7)	1.3	0.8–2.0	0.34
Ischemic heart disease	50 (15.1)	174 (10.5)	1.4	1.0–2.0	0.07
Cerebral infarction	18 (5.4)	59 (3.6)	1.4	0.8–2.3	0.34
Peripheral vascular disease	21 (6.3)	50 (3.0)	2.0	1.2–3.4	0.04
Gastrointestinal	125 (37.7)	489 (29.5)	1.3	1.1–1.6	0.02
GORD, gastritis or ulcer disease	25 (7.5)	95 (5.7)	1.3	0.8–2.1	0.30
Functional intestinal disorders	28(8.4)	66(4.0)	2.2	1.4–3.4	0.006
Musculoskeletal system and connective tissue	140 (42.2)	739 (44.6)	1.0	0.8–1.2	0.89
Inflammatory polyarthropathies	8 (2.4)	57 (3.4)	0.7	0.3–1.5	0.40

*ILA* interstitial lung abnormalities, *HR* hazard ratio, *COPD* chronic obstructive pulmonary disease, *GORD* gastro-oesophageal reflux disease

Number of participants with ILA compared with participants without ILA for receiving one of several specific diagnoses of interest at a hospital admission, outpatient clinic visits or emergency department visit. Cox regression analysis is adjusted for age, sex, BMI and pack-years. *P*-values are adjusted for multiple comparisons by the Benjamini-Hochberg method

### ILA and hospital admission rates

**Fig. 2:**
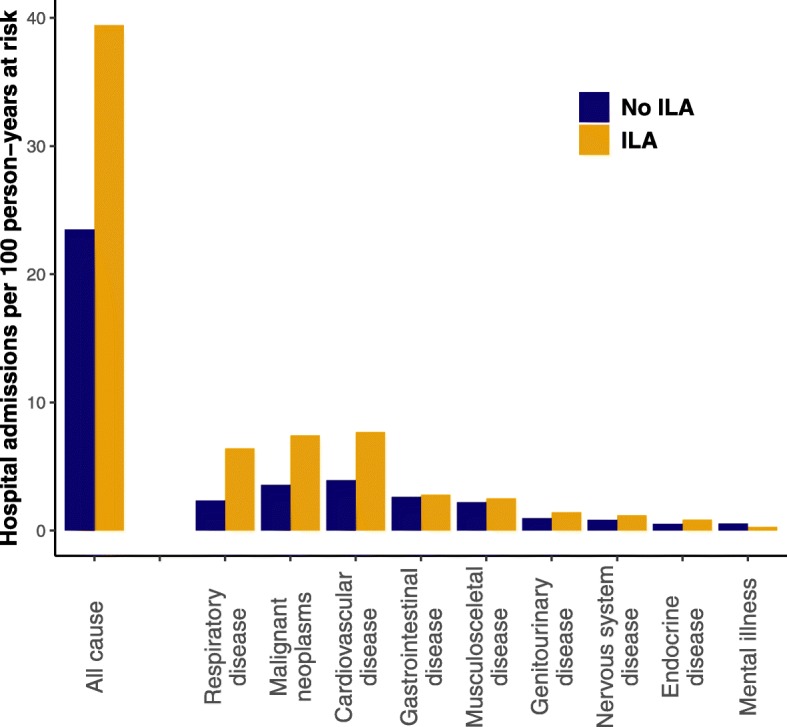
Unadjusted hospital admission rates in participants with ILA and those without ILA. ILA: interstitial lung abnormalities

**Fig. 3:**
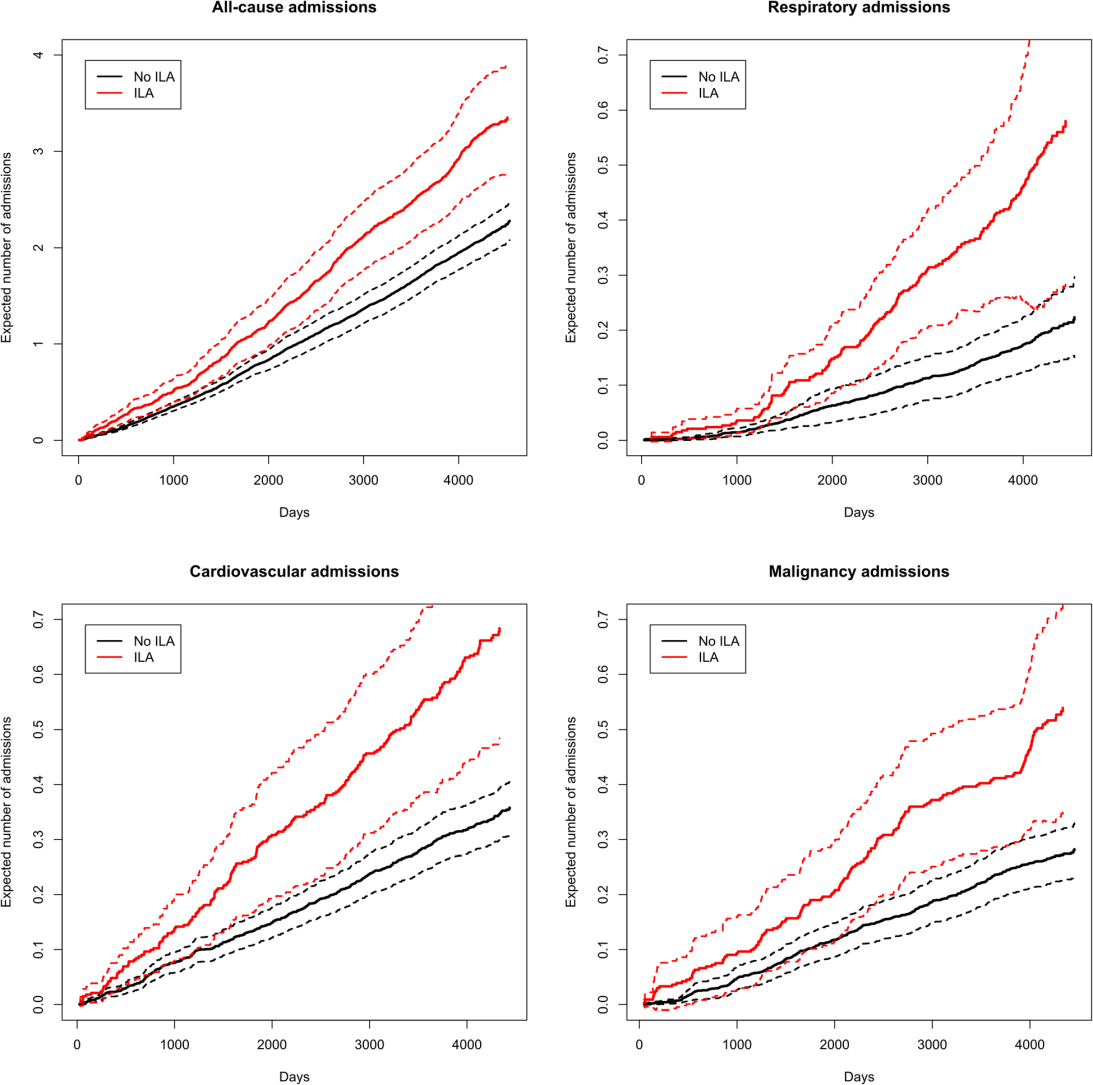
Expected number of hospital admissions in participants with ILA and without ILA respectively based on a marginal Cox proportional hazards model of a recurrent event model (hospital admissions) with gap times and a terminal event model (death). Dashed lines indicate standard errors. Note the different scales for all-cause hospital admissions and specific admissions, respectively

Participants with ILA had a higher crude mean admission rate during follow-up compared with participants without ILA (39 vs. 23 admissions per 100 person-years at risk) (Fig. 2). In a multivariate recurrent event Cox proportional hazards analysis, participants with ILA had a significantly higher hazard rate for hospital admission during short-term (1 year) follow-up (HR: 1.8, 95% CI: 1.3–2.6, *p* = 0.002) and long-term (12 years) follow-up (HR: 1.4, 95% CI: 1.2–1.7, *p* < 0.001) (Fig. 3).

After stratifying hospital admissions by discharge diagnoses, we found a significant increase in admissions due to respiratory and malignant diseases in participants with ILA (Table 3, Figs. 2 and 3, Supplementary Figure 2). Most notably, admissions for pneumonia and lung cancer were more frequent (Table 2). Hospital admissions for COPD were not associated with ILA when only considering the primary discharge diagnosis (Table 3). However, when expanding the analysis to include all discharge diagnoses (primary diagnosis and contributing diagnoses), participants with ILA had more frequent hospital admissions also with COPD (HR: 2.1, 95% CI: 1.3–3.3, *p* = 0.003). Hospital admissions with cardiovascular disease appeared to be increased in the unadjusted model (Fig. 3) but not in the multivariate model (Table 3). However, admissions for pulmonary embolism and peripheral vascular disease were consistently increased in participants with ILA (Table 3).

**Table 3:** Disease specific hospital admissions and emergency-department (ED) visits in participants with or without ILA

Disease group	HR	95% CI	Adjusted *p*-value
Respiratory disease	2.1	1.4–3.1	0.001
COPD	2.5	0.9–6.8	0.10
Pneumonia	2.1	1.5–3.0	0.001
Asthma	0.8	0.1–7.7	0.90
Pleural empyema or lung abscess	0.3	0.0–32.6	0.90
Interstitial lung disease	1.8	0.3–12.5	0.82
Malignant neoplasm	1.6	1.2–2.1	0.001
Lung cancer	2.8	1.7–4.5	0.001
Non-pulmonary cancer	1.3	0.9–1.9	0.15
Cardiovascular disease	1.4	1.0–1.8	0.10
Heart failure	2.2	1.3–4.1	0.09
Pulmonary embolism	4.9	1.6–14.7	0.01
Atrial fibrillation/atrial flutter	1.3	0.5–3.3	0.82
Ischemic heart disease	1.1	0.8–1.6	0.90
Cerebral infarction	1.5	0.8–2.7	0.43
Peripheral vascular disease	5.0	2.5–9.9	0.001
Gastrointestinal disease	1.1	0.8–1.6	0.87
GORD, gastritis or ulcer disease	0.9	0.3–2.2	0.90
Functional intestinal disorders	1.1	0.4–3.0	0.90
Diseases of the musculoskeletal system and connective tissue	1.1	0.8–1.4	0.90
Inflammatory polyarthropathies	1.5	0.9–2.4	0.95

*ILA* interstitial lung abnormalities, *HR* hazard ratio, *GORD* gastro-oesophageal reflux disease

Adjusted hazard ratios for disease specific hospital admissions and emergency-department (ED) visits in participants with ILA compared with participants without ILA. Cox regression analysis is adjusted for age, sex, BMI and pack-years. P-values are corrected multiple comparisons by the Benjamini-Hochberg method

The hazard rate of emergency department visits was similar in participants with or without ILA (HR: 1.3, 95% CI: 0.8–2.0, *p* = 0.36).

### ILA and primary care visits

The rate of visits to a primary care provider during follow-up was slightly increased in participants with ILA compared with participants without ILA (4.51 vs. 4.05 visits per person year at risk, *p* = 0.01). However, this difference was no longer significant in the multivariate model (Supplementary Figures 3 and 4).

### ILA and medicine use

Participants with ILA had a higher overall rate of collected drug prescriptions during follow-up (median 3.1 vs. 2.5 prescriptions per person year at risk, *p* = 0.009). After adjusting for potential confounders in multivariate negative binomial regression analysis, there remained an increased use of several medications, including inhalation therapy (*p* = 0.009), antibiotic therapy (*p* = 0.002) and loop diuretics (*p* = 0.008) (Table 4).

**Table 4:** Proportion of participants with at least one prescription for the listed classes of medicine

Medication	ILA	No ILA	*P*-value
Inhalation therapy	72 (22%)	316 (19%)	0.009
Antibiotic therapy	277 (83%)	1337 (81%)	0.002
Prednisolone	27 (8%)	130 (8%)	0.63
Proton pump inhibitors	111 (33%)	482 (29%)	0.07
Antithrombotic therapy	98 (30%)	361 (22%)	0.35
Antihypertensive therapy	77 (23%)	348 (21%)	0.09
Loop diuretics	27 (8%)	79 (5%)	0.008
Lipid lowering therapy	108 (33%)	517 (31%)	0.64
Antidiabetic therapy	75 (23%)	335 (20%)	0.86

*ILA* interstitial lung abnormalities

*P*-values based on negative binomial regression of the number of prescriptions, adjusted for age, sex, BMI and pack-years

## Discussion

In this 12-year long follow-up of lung cancer screening trial participants, we show an increased disease specific morbidity and healthcare utilisation in participants with ILA. This includes a more frequent diagnosis of several respiratory diseases, such as ILD, COPD, pulmonary infections, lung cancer and respiratory failure, a higher hospital admission rates, and increased use of several therapies for these diseases.

### ILA and specific diagnoses

A higher proportion of participants with ILA received a hospital diagnosis of a respiratory disease or lung cancer in the 12 years following the radiologic finding. Our results add to previous reports of increased lung cancer related mortality and to a lesser extent respiratory mortality in individuals with ILA [7, 13, 24]. However, the present study adds to the understanding of ILA by describing an increased frequency of several more specific respiratory diagnoses, such as ILD, COPD, pneumonia, pleural empyema and respiratory failure, after adjusting for age, sex, BMI and smoking status and correcting for multiple comparisons. It is not clear how the presence of ILA predisposes to the increased morbidity, but these rather unspecific radiological findings possibly reflect inflammatory, premalignant or pulmonary vascular changes. Further research is needed to specify the specific risk associated with the different types of ILA. ILA could also be the result of previous exposure to dust, gasses, infections or pneumotoxic medications, in a population already predisposed to respiratory diseases.

The association between ILA and the development of clinical ILD highlights the potential for an earlier diagnosis by recognizing ILA in a lung cancer screening setting [25]. An increased incidence of ILD has previously been shown in individuals with high-attenuation areas in the lung, a quantitative assessment of parenchymal abnormalities [24]. This association was found for community dwelling individuals, both smokers and non-smokers, while the present study is limited to long-term smokers [24]. There is thus growing evidence of an increased risk of developing ILD in people with areas of increased attenuation in the lung parenchyma, irrespective of the way these are determined. Further research is needed to identify radiological, clinical or genetic risk factors for the development of specific ILDs. In IPF, radiological findings can be visible many years before clinical disease, making screening by CT in conjunction with lung cancer screening an attractive option [10, 25]. Considering that IPF is more common in smokers and older people, who also are the candidate population for lung cancer screening, some cases of subclinical IPF could be detected as incidental findings from the CT scans in a lung cancer screening program [25, 26].

The association between ILA and clinically diagnosed COPD during long-term follow-up is intriguing, as it contradicts some earlier cross-sectional and case control findings but is supported by others [6, 27, 28]. Our results could be due to the study population derived from a lung cancer screening trial, the longitudinal follow-up or a different definition of clinical disease. First, we included long-term smokers from a lung cancer screening trial and followed them for up to 12 years, which may affect the incidence of COPD as both age and tobacco exposure are known risk factors of COPD. Second, we registered all hospital diagnoses of COPD, with no regard to disease severity. Previous studies found that people with ILA had a decrease in their odds of having COPD of stage 2 or higher [6, 29]. It has previously been suggested that ILA can be a marker of susceptibility to smoking related lung injury, which is supported by our findings during longitudinal follow-up [28].

The marked increase in pulmonary infections in participants with ILA was confirmed by an increased use of antibiotic therapy. This association could have several explanations. First, patients with ILA were older and more frequently active smokers, and thus more susceptible to pneumonia [30]. Second, the higher frequency of COPD, a disease associated with pulmonary infections and exacerbations, in participants with ILA would lead to an expected increase in these infections.

In line with the general increase in respiratory disease, a hospital diagnosis of respiratory failure was twice as frequent in participants with ILA compared with those without ILA. A previous study has shown that critically ill patients with sepsis, who had ILA on chest CT scans taken within 1 week prior to ICU admission were more likely to develop acute respiratory distress syndrome [31]. We supplement these findings with longitudinal follow-up showing that a finding of ILA also increases the long-term risk of developing respiratory failure.

### ILA and hospital admission rates

Participants with ILA had a higher rate of hospital admissions during both short-term and long-term follow-up. Hospital admission rates are measures of morbidity that are highly relevant to both patients and healthcare systems, and are a recommended outcome for clinical trials of IPF alongside mortality [32, 33]. Our results thus highlight the clinical and economical importance of ILA as incidental findings [13].

The most pronounced increase in hospital admissions for participants with ILA was found for respiratory and malignant causes, which corresponds with our finding of an increased incidence of these diseases in participants with ILA. The increased rate of hospital admissions with pulmonary embolism and peripheral vascular disease in participants with ILA was more surprising. Venous thromboembolic disease is associated with several ILDs, including lung fibrosis, sarcoidosis and IPF [34–37]. To our knowledge, we present for the first time an increase in pulmonary embolism morbidity also in individuals with ILA. The higher prevalence of malignancy, which is a known risk factor for thromboembolic disease, in participants with ILA could be a possible explanation. Alternatively, ILA and thromboembolic disease could share common, and possibly unknown, risk factors.

The gastrointestinal disorders which were more prevalent in patients with ILA did not result in increased hospitalisation rates, which could be expected from these diagnoses. They are likely to be handled in an outpatient setting rather than causing hospital admissions.

### Limitations

There is a lack of standardization of ILA across different studies which makes comparisons difficult [38]. Previous studies have analysed ILA in different ways. Many studies have excluded indeterminate ILA in their analysis [6, 8, 11, 24], some have graded the extent of the abnormalities [4], some have studied high attenuation areas on CT rather than visually defined ILA [5, 24, 39], and some have identified specific patterns [2, 4]. Furthermore, some studies have supplemented low dose CT findings with HRCT to exclude false positive ILA [4, 6]. In contrast to other reports, we did not code any findings as ‘indeterminate’ or ‘equivocal’ but limited the analysis to a dichotomous variable of ‘ILA’ or ‘No ILA’. Other studies report a large variation in the proportion of indeterminate ILA subjects from around 12–59% [7, 8]. Our conclusions are potentially affected by including less severe indeterminate findings in the ‘exposed’ group. However, previous studies have found associations between interstitial features in smokers and reduced lung function, worse quality of life and increased mortality, even in participants who did not have visually defined ILA or who were classified as indeterminate for ILA [7, 28]. We also relied on qualitative descriptors of ILA rather than quantitative measures which reduces the repeatability of our findings due to the known interobserver variability of radiologic findings even among experienced radiologists [40]. These differences must be taken into account when interpreting our results and comparing them to other studies.

We report a higher prevalence of ILA compared with other cohorts [6–8, 24]. This could be due to the fact, that the population was derived from a lung cancer screening trial of long-term smokers. The higher prevalence of ILA could also be due to the reading method used. In the present study, participants were classified as having ILA if at least one observer scored it as such. A more rigorous sequential reading method, could have reduced the number of definite ILA findings. Finally, the classification used where participants were scored as ‘ILA’ or ‘No ILA’ without any ‘indeterminate’ category, could have lead to a higher prevalence of ILA in our cohort.

The data on the specific contact diagnoses were only available for secondary care contacts (hospital admissions, outpatient clinic visits and emergency department visits). This could lead to potentially underestimating the prevalence of certain diagnoses (i.e. COPD or pneumonia) for participants treated exclusively in primary care. However, for many diagnoses of interest, such as lung cancer and ILD, participants would be expected to be diagnosed in secondary care.

The participants in the present study were mostly white northern Europeans. The generalizability to other ethnicities remains to be determined.

## Conclusions

Individuals with ILA have an increased morbidity related to several respiratory diseases, including ILD, COPD, pulmonary infections, lung cancer and respiratory failure. This should be taken into account in the healthcare plans of this population.

## Supplementary information

**Additional file 1: Supplementary Figure 1.** Flowchart of inclusion of patients in the follow-up study. DLCST. Danish lung cancer screening trial. **Supplementary Figure 2.** Expected number of hospital admissions in participants with ILA and without ILA respectively based on a marginal Cox proportional hazards model of a recurrent event model (hospital admissions) with gap times and a terminal event model (death). Dashed lines indicate standard errors. Note the different scales. DJ: respiratory disease, DI: cardiovascular disease, DC: malignant disease, DK: gastrointestinal disease, DM: diseases of the musculoskeletal system, DS and DT: injury and poisoning, DN: genitourinary disease, DG: nervous system disease**. Supplementary Figure 3.** Expected number of visits to a general practitioner in participants with ILA and without ILA respectively based on a marginal Cox proportional hazards model of a recurrent event model (GP visits) and a terminal event model (death). **Supplementary Figure 4.** Unadjusted GP-visit rates in participants with ILA and participants without ILA. ILA: interstitial lung abnormalities. **Supplementary Table 1.** ICD10 codes for the classification of contact diagnoses for the analysis of development of disease and hospital admission rates. **Supplementary Table 2**. ATC codes used to classify medicine groups for the analysis of medicine use. **Supplementary Table 3**. Number of the specific ILA findings on the CT scans. Some scans had more than one type of ILA. **Supplementary Table 4**. Risk of receiving a specific diagnosis during follow-up in participants with and without ILA, respectively. Cox regression analysis is adjusted for age, sex, BMI, pack-years and FEV_1_. ILA: interstitial lung abnormalities, HR: hazard ratio, COPD: chronic obstructive pulmonary disease, GORD: gastro-oesophageal reflux disease.

## Data Availability

The datasets used and/or analysed during the current study are available in an anonymized form from the corresponding author on reasonable request.

## Abbreviations

BMI: Body mass index
COPD: Chronic obstructive pulmonary disease
CT: Computed tomography
DLCST: Danish lung cancer screening trial
FEV_1_: Forced expiratory volume in one second
FVC: Forced vital capacity
ILA: Interstitial lung abnormalities
ILD: Interstitial lung disease
IPF: Idiopathic pulmonary fibrosis
