# Supplementary material for: Increased respiratory morbidity in individuals with interstitial lung abnormalities
Source: BMC Pulm Med. 2020 Mar 19;20:67. doi: 10.1186/s12890-020-1107-0 (PMC7081690; doi:10.1186/s12890-020-1107-0)
Supplement: Supplementary file 1 — Additional file 1: Supplementary Figure 1. Flowchart of inclusion of patients in the follow-up study. DLCST. Danish lung cancer screening trial. Supplementary Figure 2. Expected number of hospital admissions in participants with ILA and without ILA respectively based on a marginal Cox proportional hazards model of a recurrent event model (hospital admissions) with gap times and a terminal event model (death). Dashed lines indicate standard errors. Note the different scales. DJ: respiratory disease, DI: cardiovascular disease, DC: malignant disease, DK: gastrointestinal disease, DM: diseases of the musculoskeletal system, DS and DT: injury and poisoning, DN: genitourinary disease, DG: nervous system disease. Supplementary Figure 3. Expected number of visits to a general practitioner in participants with ILA and without ILA respectively based on a marginal Cox proportional hazards model of a recurrent event model (GP visits) and a terminal event model (death). Supplementary Figure 4. Unadjusted GP-visit rates in participants with ILA and participants without ILA. ILA: interstitial lung abnormalities. Supplementary Table 1. ICD10 codes for the classification of contact diagnoses for the analysis of development of disease and hospital admission rates. Supplementary Table 2. ATC codes used to classify medicine groups for the analysis of medicine use. Supplementary Table 3. Number of the specific ILA findings on the CT scans. Some scans had more than one type of ILA. Supplementary Table 4. Risk of receiving a specific diagnosis during follow-up in participants with and without ILA, respectively. Cox regression analysis is adjusted for age, sex, BMI, pack-years and FEV1. ILA: interstitial lung abnormalities, HR: hazard ratio, COPD: chronic obstructive pulmonary disease, GORD: gastro-oesophageal reflux disease. [file 12890_2020_1107_MOESM1_ESM.docx]

## Supplementary material


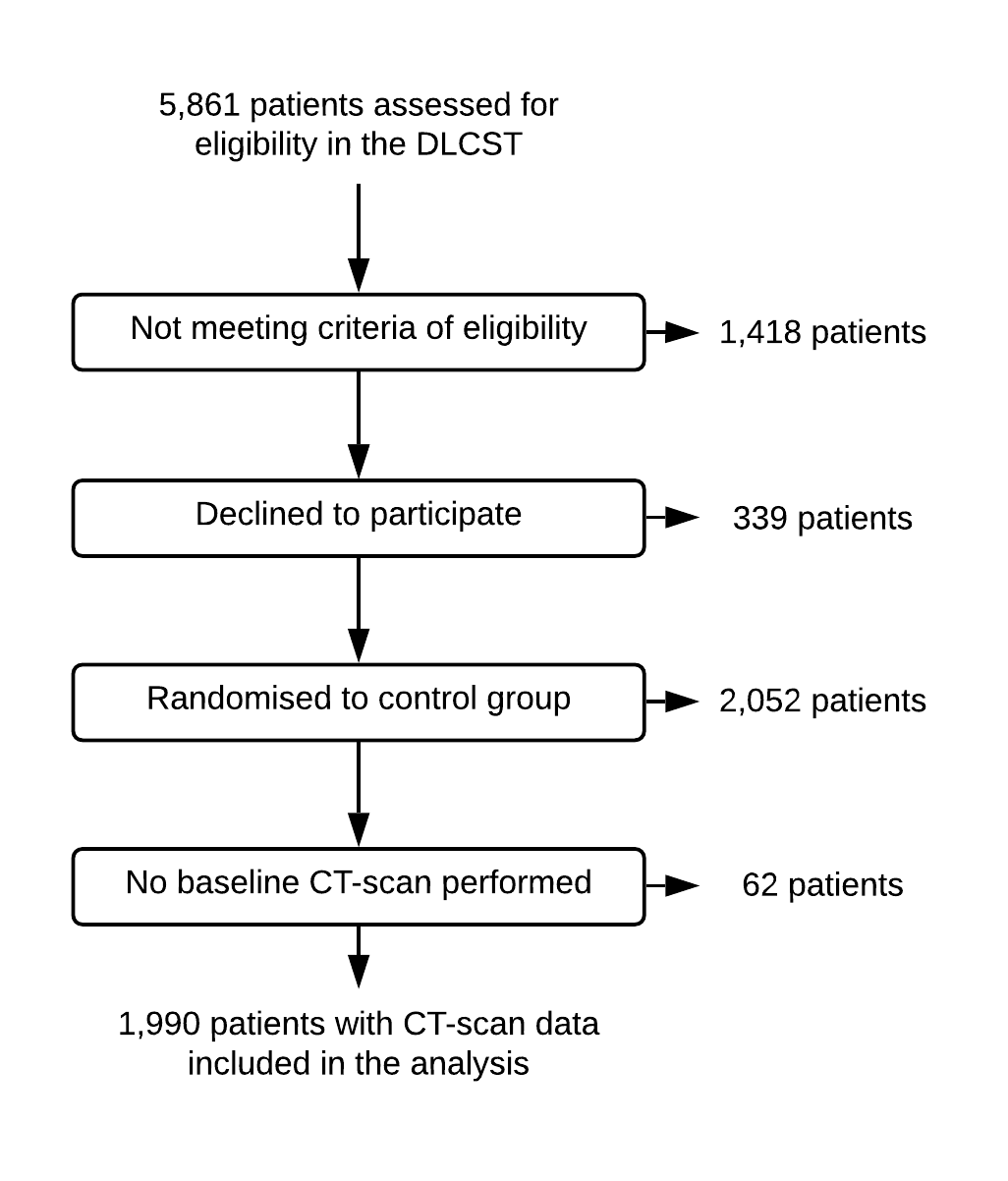


Supplementary figure 1: Flowchart of inclusion of patients in the follow-up study. DLCST. Danish lung cancer screening trial.


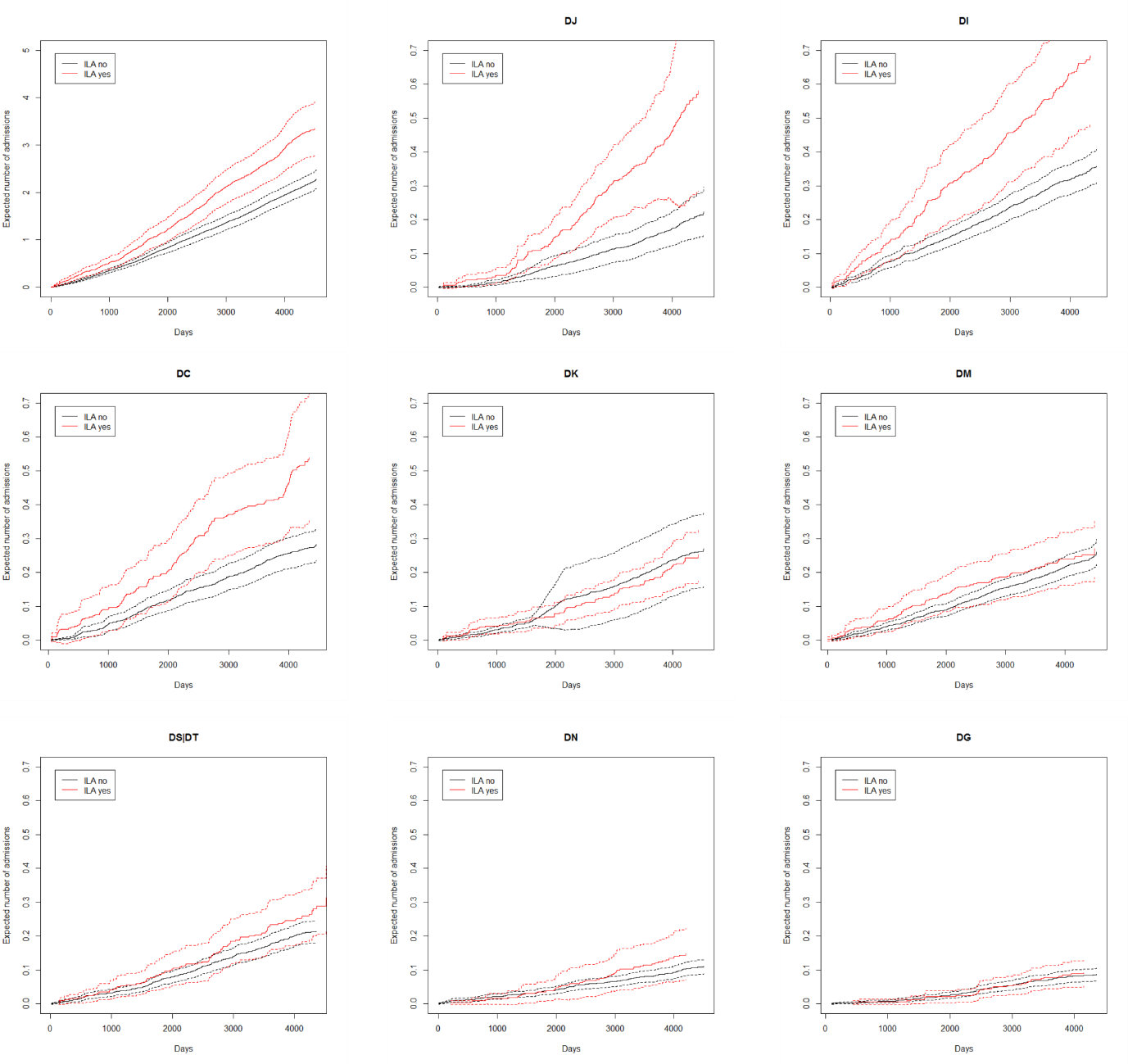


Supplementary figure 2: Expected number of hospital admissions in participants with ILA and without ILA respectively based on a marginal Cox proportional hazards model of a recurrent event model (hospital admissions) with gap times and a terminal event model (death). Dashed lines indicate standard errors. Note the different scales. DJ: respiratory disease, DI: cardiovascular disease, DC: malignant disease, DK: gastrointestinal disease, DM: diseases of the musculoskeletal system, DS and DT: injury and poisoning, DN: genitourinary disease, DG: nervous system disease


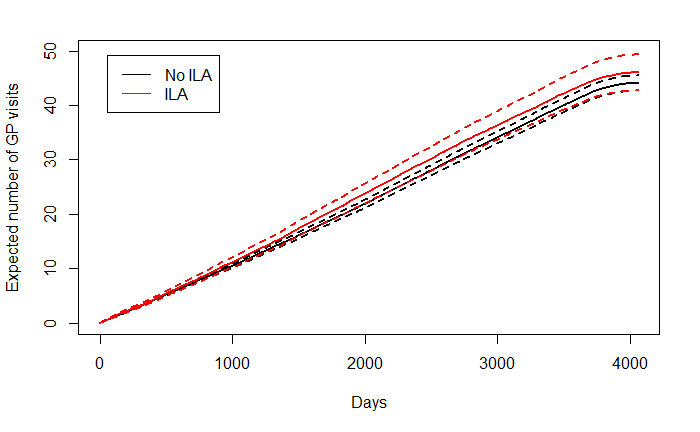


Supplementary figure 3: Expected number of visits to a general practitioner in participants with ILA and without ILA respectively based on a marginal Cox proportional hazards model of a recurrent event model (GP visits) and a terminal event model (death).


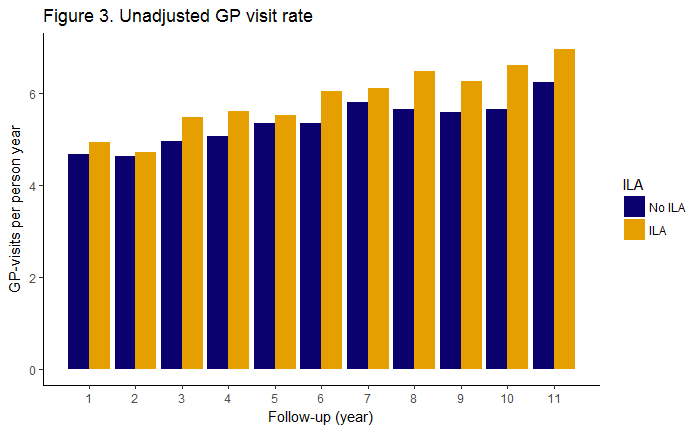


Supplementary figure 4: Unadjusted GP-visit rates in participants with ILA and participants without ILA. ILA: interstitial lung abnormalities

| **Disease group** | **ICD-10 codes** |
| --- | --- |
| Respiratory disease | DJ00–DJ99 |
| COPD | DJ40–DJ44 |
| Pneumonia | DJ09–DJ18 |
| Asthma | DJ45 |
| Pleural empyema or lung abscess | DJ85–DJ86 |
| Interstitial lung disease | DJ84, DJ60–DJ64, DJ67 |
| Respiratory failure | DJ96 |
|  |  |
| Cardiovascular disease | DI00–DI99 |
| Heart failure | DI50 |
| Pulmonary embolism | DI26 |
| Atrial fibrillation/atrial flutter | DI48 |
| Ischemic heart disease | DI20–DI25 |
| Cerebral infarction | DI63 |
| Peripheral vascular disease | DI73 |
|  |  |
| Malignant neoplasm | DC00–DC99 |
| Lung cancer | DC34 |
| Non-pulmonary cancer | DC00–DC29, DC40–DC99 |
|  |  |
| Gastrointestinal disease | DK00–DK99 |
| GERD, gastritis or ulcer disease | DK20–DK30 |
| Functional intestinal disorders | DK59 |
|  |  |
| Diseases of the musculoskeletal system and connective tissue | DM00–DM99 |
| Inflammatory polyarthropathies | DM04–DM14 |
|  |  |
| **Supplementary table 1:** ICD10 codes for the classification of contact diagnoses for the analysis of development of disease and hospital admission rates. | |

| **Medicine group** | **ATC code** |
| --- | --- |
| Inhalation therapy | R03A, R03B |
| Antibiotic therapy | J01 |
| Prednisolone | H02AB06 |
| Proton pump inhibitors | A02BC |
| Antithrombotic therapy | B01A |
| Antihypertensive therapy | C02 |
| Loop diuretics | C03C |
| Lipid lowering agents | C10 |
| Antidiabetics | A10 |
|  |  |
| **Supplementary table 2:** ATC codes used to classify medicine groups for the analysis of medicine use. | |

| **CT finding** | **N (%)** |
| --- | --- |
| All ILA | 332 (16.7%) |
| Ground glass | 115 (5.8%) |
| Nodular pattern | 155 (7.8%) |
| Reticular pattern | 162 (8.1%) |
| Honeycombing | 12 (0.6%) |
|  |  |
| **Supplementary table 3:** Number of the specific ILA findings on the CT scans. Some scans had more than one type of ILA. | |

| Diagnosis | ILA (%) | No ILA (%) | HR | 95% CI | P-value |
| --- | --- | --- | --- | --- | --- |
| Respiratory | 116 (34.9) | 361 (21.8) | 1.44 | 1.16-1.79 | < 0.001 |
| COPD | 55 (16.6) | 159 (9.6) | 1.24 | 0.90-1.71 | 0.190 |
| Pneumonia | 56 (16.9) | 126 (7.6) | 1.74 | 1.25-2-41 | < 0.001 |
| Asthma | 4 (1.2) | 47 (2.8) | 0.36 | 0.13-1.02 | 0.054 |
| Pleural empyema or lung abscess | 5 (1.5) | 3 (0.2) | 5.55 | 1.24-24.77 | 0.025 |
| Interstitial lung disease | 8 (2.4) | 8 (0.5) | 4.56 | 1.67-12.49 | 0.003 |
| Respiratory failure | 25 (7.5) | 61 (3.7) | 2.02 | 1.09-3.75 | 0.025 |
|  |  |  |  |  |  |
| Malignant neoplasm | 93 (28.0) | 317 (19.1) | 1.33 | 1.05-1.68 | 0.019 |
| Lung cancer | 39 (11.7) | 71 (4.3) | 2.36 | 1.57-3.53 | < 0.001 |
| Non-pulmonary cancer | 67 (20.2) | 266 (16.0) | 1.16 | 0.89-1.53 | 0.280 |
|  |  |  |  |  |  |
| Cardiovascular | 157 (47.3) | 652 (39.3) | 1.18 | 0.99-1.41 | 0.072 |
| Heart failure | 19 (5.7) | 47 (2.8) | 1.37 | 0.78-2.39 | 0.270 |
| Pulmonary embolism | 8 (2.4) | 22 (1.3) | 1.79 | 0.78-4.12 | 0.170 |
| Atrial fibrillation/atrial flutter | 27 (8.1) | 95 (5.7) | 1.26 | 0.82-1.96 | 0.296 |
| Ischemic heart disease | 50 (15.1) | 174 (10.5) | 1.36 | 0.98-1.87 | 0.065 |
| Cerebral infarction | 18 (5.4) | 59 (3.6) | 1.25 | 0.73-2.16 | 0.420 |
| Peripheral vascular disease | 21 (6.3) | 50 (3.0) | 1.72 | 1.01-2.92 | 0.044 |
|  |  |  |  |  |  |
| Gastrointestinal | 125 (37.7) | 489 (29.5) | 1.29 | 1.06-1.58 | 0.012 |
| GORD, gastritis or ulcer disease | 25 (7.5) | 95 (5.7) | 1.27 | 0.81-1.99 | 0.30 |
| Functional intestinal disorders | 28(8.4) | 66(4.0) | 2.11 | 1.34-3.31 | 0.001 |
|  |  |  |  |  |  |
| Musculoskeletal system and connective tissue | 140 (42.2) | 739 (44.6) | 1.00 | 0.84-1.21 | 0.940 |
| Inflammatory polyarthropathies | 8 (2.4) | 57 (3.4) | 0.72 | 0.34-1.52 | 0.380 |
|  |  |  |  |  |  |
| **Supplementary table 4:** Risk of receiving a specific diagnosis during follow-up in participants with and without ILA, respectively. Cox regression analysis is adjusted for age, sex, BMI, pack-years and FEV_1_. ILA: interstitial lung abnormalities, HR: hazard ratio, COPD: chronic obstructive pulmonary disease, GORD: gastro-oesophageal reflux disease | | | | | |
